# Quinoa Improves Non‐Alcoholic Fatty Liver Disease by Regulating the Ras‐PLD Pathway to Activate Autophagy

**DOI:** 10.1002/fsn3.71233

**Published:** 2025-11-25

**Authors:** Yuelin Zhang, Zhengting Liang, Jiaxian Liu, Xianjie Zhen, Ruijie Liu, Xiaohong Luo, Guangjian Jiang

**Affiliations:** ^1^ School of Traditional Chinese Medicine Beijing University of Chinese Medicine Beijing China; ^2^ School of Traditional Chinese Medicine Xinjiang Medical University Xinjiang China; ^3^ Zhongli Science and Technology Limited Company Beijing China; ^4^ Gansu Pure High‐Land Agricultural Science and Technology Limited Company Lanzhou China; ^5^ Xinjiang Tianrun Dairy Co., Ltd. Urumqi China

**Keywords:** gut microbiota, molecular docking, NAFLD, network pharmacology, quinoa

## Abstract

The beneficial effects of quinoa on metabolic diseases have been extensively investigated. In this context, we sought to study the efficacy of quinoa in a nonalcoholic fatty liver disease (NAFLD) mouse model and its underlying mechanism of action. Male ICR mice were fed a high‐fat diet for 12 weeks to establish a NAFLD model, followed by 6 weeks of quinoa intervention. The mechanism by which quinoa improves NAFLD was explored using network pharmacology analysis and transcriptome sequencing analysis. Finally, combined with in vivo experiments for verification. Quinoa significantly improved liver damage, abnormal glucose and lipid metabolism, and insulin resistance in NAFLD mice. The results of network pharmacology and transcriptomics indicate that the Ras‐PLD signaling pathway and autophagy are key pathways. Quinoa significantly regulates the expression of Ras, Raf, Mek, Erk, and autophagy‐related proteins in liver tissue. Meanwhile, quinoa regulates the expression of HNF4A, ACOX2, and glucagon in the liver and pancreas. Our results suggest that quinoa may improve NAFLD by inhibiting the Ras‐PLD signaling pathway and activating autophagy, regulating glucose and lipid metabolism and insulin resistance.

## Graphical abstract

Quinoa alleviates liver injury, glucose/lipid metabolic disorders, and insulin resistance in high‐fat diet‐induced NAFLD mice. Mechanistically, it acts by inhibiting the Ras‐PLD signaling pathway, activating autophagy, and regulating key related protein/gene expressions, as validated via network pharmacology, transcriptomics, and in vivo experiments.

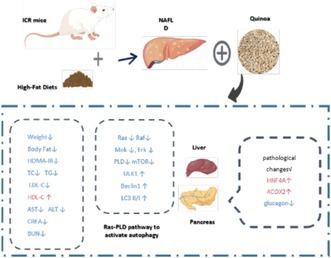

## 1 Introduction

Nonalcoholic fatty liver disease (NAFLD) is generally initiated by excess fat accumulation in the liver and can progress to inflammation, fibrosis, advanced fibrosis, and cirrhosis (Koutoukidis et al. 2019; Townsend and Newsome 2016). Approximately one quarter of the global population currently suffers from this disease, and its prevalence continues to increase (Riazi et al. 2022). Obesity is the main cause of NAFLD, which is associated with a poor prognosis (Younossi et al. 2018; Polyzos et al. 2019). Moreover, current research on the pathogenesis of NAFLD involves metagenomics, metatranscriptomics, metabolomics, and microbiology. Quinoa ( *Chenopodium quinoa* Willd.) is an ancient crop originating from the Andes Mountains of South America that is recognized as a good food supplement owing to its nutraceutical role (Vega‐Gálvez et al. 2010). As it is more resistant to harsh environmental stresses than other cereals, quinoa is regarded as a crop with the potential to help ensure food security and agricultural diversification, and is thus widely cultivated in several countries (Pulvento and Bazile 2023). In recent years, the popularity of quinoa seeds has increased owing to reports of their health benefits and super‐food properties (Yao et al. 2024; Zhong et al. 2023; Sun et al. 2025). Several studies, including ours, have shown that quinoa positively modulates metabolic diseases in various ways, including by regulating gut flora (An et al. 2021; Noratto et al. 2019). Although the efficacy of quinoa in alleviating NAFLD has been extensively demonstrated, including improved hepatic steatosis and oxidative stress state among others, additional studies on its molecular mechanisms are needed (Song et al. 2021).

Network pharmacology is a systemic methodology used to study the mechanisms of drugs at the systemic level and decipher complex biological pathways, and is a valuable tool for elucidating the efficiency of complex natural products (Kibble et al. 2015) (Yu et al. 2024). Transcriptomics is a discipline that studies gene transcription and transcriptional regulation at the global level, analyzing dynamic changes in gene expression and transcriptional diversity (Wang et al. 2009). A comprehensive analysis based on network pharmacology and transcriptomics can better understand complex biological processes and help identify key factors (Zhao et al. 2023; Yan et al. 2024).

Therefore, it is necessary to develop a new method and concept to reveal the effects of quinoa in NAFLD. In this study, we aim to elucidate the effects of the quinoa diet on NAFLD mice, as well as the regulation of related indicators such as glucose, lipid metabolism, and insulin resistance, in order to reveal its potential mechanisms.

## 2 Experimental Section

### 2.1 Animals

Offspring of male ICR mice whose sires were obese and dams were normal (SCXK (Jing) 2016–0011) were purchased from Beijing Vital River Laboratory Animal Technology Limited Company (Beijing, China). Prior to the start of the experiment, all mice were acclimatized and fed in a standard animal feeding room for 3 days. The mice were fed an ordinary diet or a high‐fat diet (HFD). The diet was purchased from Beijing HFK Bioscience Co. LTD (Beijing, China), and HFD was 60% fat. The rearing environment and experimental diet were maintained as described previously (Wang et al. 2022). All animal experiments were approved by the Institutional Animal Care and Use Committee of Beijing University of Chinese Medicine and conformed to current animal welfare and approved guidelines (permission number: BUCM‐2019041501‐200J).

### 2.2 Construction of NAFLD Mouse Model and Interventions

A total of 20 male ICR mice were fed a normal diet for 4 weeks and then randomly assigned to a control group (*n* = 5) and an obese group (*n* = 15), which were fed normal and high‐fat diets, respectively, for 12 weeks. Mice in the obese group were randomly divided into an NAFLD group, quinoa for random time (QRT) group, and quinoa for fixation time (QFT) group, with five mice in each group, and the intervention was initiated. Mice in the control group were fed a normal diet, whereas those in the NAFLD and intervention groups were fed a high‐fat diet. Mice in the QRT group were fed quinoa daily and had no time limit, whereas those in the QFT group were fed 2 g/piece of quinoa per mouse at a fixed time of the day (8:30 a.m.). Quinoa diets were purchased from Shanxi Hao Chen Biological Technology Co. Ltd. The intervention lasted for 6 weeks. During the intervention phase, body weight and blood glucose were measured at the end of each week. Body fat was measured using a small animal body composition analyzer (EMR‐170‐S) at the end of the sixth week, and body fat and lean content were expressed as a percentage of total body weight.

### 2.3 Biochemical Analysis

Blood was collected from mouse eyeballs and used for further analysis. Blood glucose (GLU), serum alanine transaminase (ALT), serum aspartate transaminase (AST), triglycerides (TG), cholesterol (TC), high‐density lipoprotein cholesterol (HDL‐C), low‐density lipoprotein cholesterol (LDL‐C), serum creatinine (CREA), and blood urea nitrogen (BUN) were measured using an automatic biochemical analyzer (Hitachi, Japan). Insulin levels were detected by radioimmunoassay. Homeostatic model assessment for insulin resistance (HOMA‐IR) was calculated (fasting insulin in μIU/mL × fasting glucose in mmol/l/22.5) and was used as a surrogate marker of insulin resistance (Wallace et al. 2004).

### 2.4 Hematoxylin–Eosin (HE) Staining

The liver, pancreas, white adipose tissues and inguinal adipose tissue were harvested from each mouse and fixed in formalin for 24 h, after which gradient dehydration was performed and tissues were embedded in paraffin for sectioning. The sections were subsequently stained with hematoxylin and eosin and mounted on slides in resin. Images were captured to evaluate pathological changes.

### 2.5 Immunohistochemistry (IHC) and Western Blotting (WB) Analysis

For IHC, the liver tissues were harvested, sectioned (5 μm), and fixed in 4% paraformaldehyde using conventional procedures. IHC was performed using rabbit ERK1/2 (1:150; ABclonal), Phospho ERK1/2 (p‐ERK1/2) (1:150; ABclonal), and N/H/K‐RAS (1:200; ImmunoWay), Raf1 (1:150; ABclonal), MEK‐1 (1:200; UpingBio), p62 (1:500; Servicebio), Beclin1 (1:100; Selleck), ULK1 (1:100; Wanlei), mTOR (1:100; Selleck). Image J software was used to assess the results and calculate the average optical density. Use WB to detect the expression of HNF4A, ACOX2, and glucagon protein in the liver and pancreas. WB was performed using first antibodies against rabbit HNF4A (1:2000; ABclone), rabbit ACOX2 (1:2000; ABclone), rabbit anti glucagon (1:20000; ABclone), rabbit anti LC3B (1:1000; Selleck) and rabbit anti β‐actin (1:5000; Biorigin). Use Image J to analyze protein expression bands and normalize them to the intensity of the corresponding bands of β‐actin. All WB experiments were conducted using three samples per group.

### 2.6 Network Pharmacology Analysis

We screened prospective targets by intersecting quinoa‐ and NAFLD‐related targets. A literature review of PubMed and CNKI was performed to determine the chemical composition of quinoa. Chemical structures were obtained from the PubChem database (https://pubchem.ncbi.nlm.nih.gov/pccompound/), chemical source network (www.Chemsrc.com), and a chemical professional database (www.organchem.csdb.cn). The obtained chemical structure was introduced into the SwissADME platform (http://www.swissadme.ch/), with two or more gastrointestinal absorption as “High” and five types of pharmacodynamic prediction (Lipinski, Ghose, Veber, Egan, Muegge) as “Yes” as potential active ingredients of quinoa. The key word “NAFLD” was used in the PharmGkb, OMIM, GeneCards, Drugbank, and TTD databases to search for related targets. These targets were then standardized using the UniProt database (https://www.uniprot.org/), and UniProt ID genes without human samples were removed.

Quinoa and NAFLD disease target data were imported together to a Venn diagram drawing website (http://bioinformatics.psb.ugent.be/webtools/Venn/), and the intersection of the drug and disease targets was obtained; the intersection genes in the Venn diagram output represented the potential targets of quinoa to treat NAFLD. The intersection genes were imported into the STRING database (https://string‐db.org/) to build a protein–protein interaction (PPI) network, and the confidence score was set to ≥ 0.7. After protein interaction analysis, the resulting file was exported using the Cytoscape software, and a core target map of the protein interaction network was created. Gene ontology (GO)–biological process function and Kyoto Encyclopedia of Genes and Genomes (KEGG) pathway enrichment analyses were performed using the KOBAS (http://kobas.cbi.pku.edu.cn/), DAVID (https://david‐d.ncifcrf.gov/), and Metascape (http://metascape.org) databases.

### 2.7 Transcriptome Sequencing Analysis

Transcriptome profiling of the mouse liver tissues was performed by RNA‐Seq. Three biological replicates were made for each group transcriptomic comparison. RNA integrity was assessed using the RNA Nano 6000 Assay Kit of the Bioanalyzer 2100 system (Agilent Technologies, CA, USA). Total RNA was used as input material for the RNA sample preparations, and mRNA was purified from total RNA using poly‐T oligo‐attached magnetic beads. Subsequently, double‐stranded cDNA was constructed and subsequently used to establish the cDNA library, and the library fragments were purified with AMPure XP system. At last, PCR products were purified (AMPure XP system) and library quality was assessed on the Agilent Bioanalyzer 2100 system. The clustering of the index‐coded samples was performed on a cBot Cluster Generation System using TruSeq PE Cluster Kit v3‐cBot‐HS (Illumia) according to the manufacturer's instructions. After cluster generation, the library preparations were sequenced on an Illumina Novaseq platform and 150 bp paired‐end reads were generated. After quality control, index of the reference genome was built using Hisat2 v2.0.5 and paired‐end clean reads were aligned to the reference genome using Hisat2 v2.0.5. Differential expression analysis of two groups was performed using the DESeq2 R package (1.20.0). Finally, we used clusterProfiler R package to test the statistical enrichment of differential expression genes in KEGG pathways. Using GSEA software (version 3.0) to detect differential gene expression pathways between groups.

### 2.8 Real‐Time Quantitative Reverse Transcription PCR (RT‐qPCR)

Total RNA was extracted from liver tissue using Trizol, and then reverse transcribed into cDNA using HiScript II Q RT SuperMix. Perform RT‐qPCR using Taq Pro Universal SYBR qPCR premix. The primers were designed and synthesized by Hunan Aikerui Bioengineering Co. Ltd. The upstream primer sequence of ras is CCTACCGGAAACAGGTGGTC, and the downstream primer sequence is GGTCCCGCATGGCACTATAC, with an amplification product length of 93 bp; the upstream primer sequence of Raf1 is TTCTTTCCCAATGCGTCGGA, and the downstream primer sequence is GGAGTGGACGTTGACCTCTG, with an amplification product length of 153 bp; the upstream primer sequence of Mek is GAGTGTTCGGAGTGGACCTG, and the downstream primer sequence is CTCCAAATTCCTTCTTCCAGTTGC, with an amplification product length of 139 bp; the upstream primer sequence of ERK is TTGCTTTCTCTCCCGCACAA, and the downstream primer sequence is CTGCTCCAGGTATGGGTGG, with an amplification product length of 156 bp; the upstream primer sequence of Pld1 is GGGAACGCTCTACAGGCAAT, and the downstream primer sequence is AGGTTTCCTTCCAGCTCTGC, with an amplification product length of 158 bp; the upstream primer sequence of β‐actin is CAGCCTTCCTTCTTGGGTAT, and the downstream primer sequence is AGGTCTTTACGGATGTCAACG, with an amplification product length of 95 bp. The relative expression of mRNA was quantified using 2^−ΔΔ*CT*^ with β‐actin as an internal reference.

### 2.9 Statistical Analysis

Data were analyzed using SPSS 20.0 and are expressed as means ± standard deviation. One‐way analysis of variance or non‐parametric tests were used for general data based on the normality test, and the least significant difference method was used for comparisons between groups. Graphs were drawn using GraphPad Prism (version 8.0). Related statistical methods were performed as previously described (Yu et al. 2024).

## 3 Results

### 3.1 Effects of Quinoa on Weight‐Reducing and Metabolic Parameters of NAFLD Mice

**FIGURE 1:**
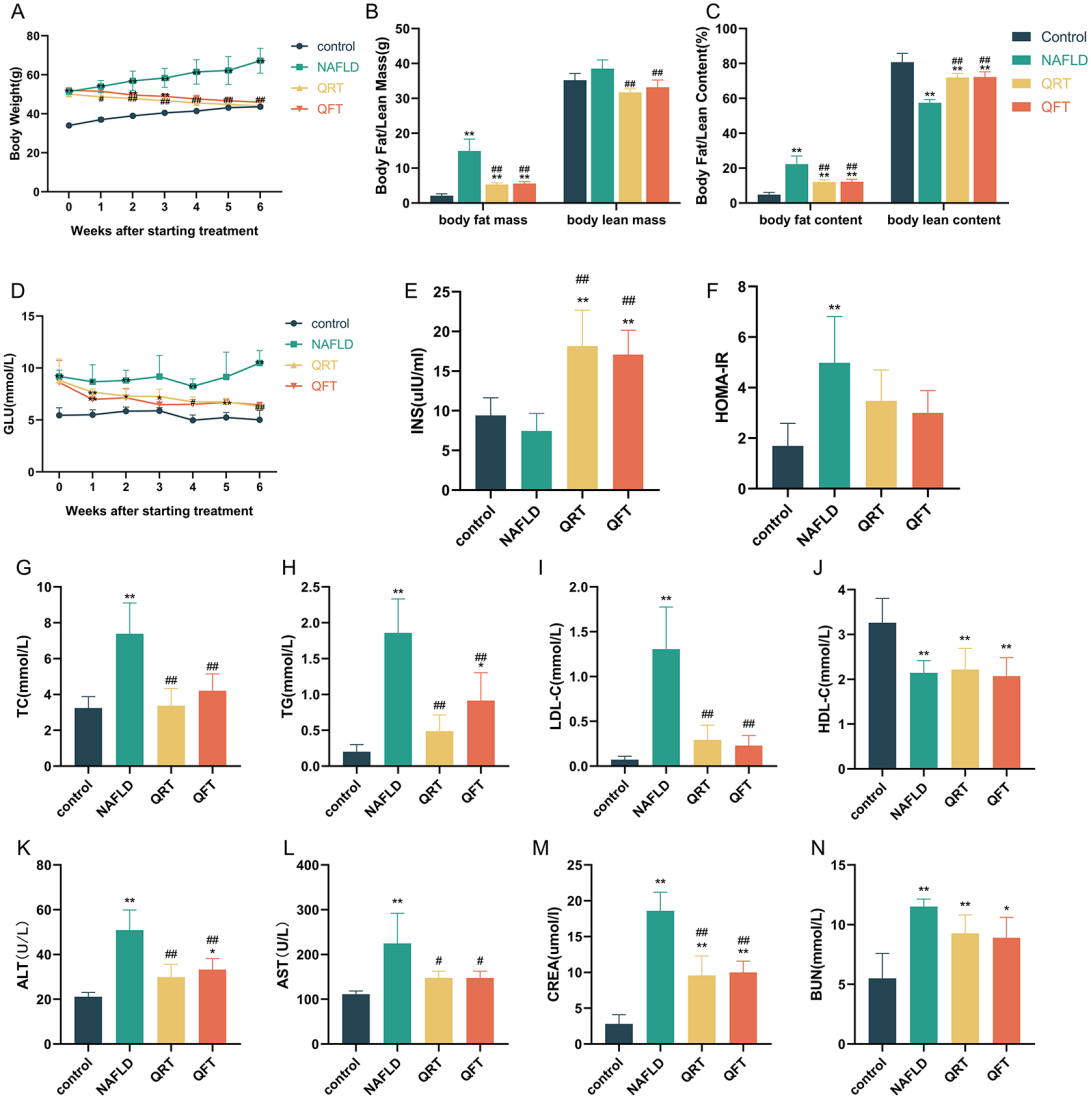
Effects of quinoa on weight‐reducing and metabolic parameters of NAFLD mice. (A) Changes of body weight. (B) Changes of body fat/lean mass. (C) Changes of body fat/lean content. (D) Changes of fasting blood glucose. (E) Changes of the insulin level. (F) Changes of the HOMA‐IR level. (G) TC; (H) TG; (I) LDL‐C; (J) HDL‐C; (K) ALT; (L) AST; (M) CREA; (N) BUN. Values are expressed as the mean ± SD of five mice in each group. *, *p* < 0.05; **, *p* < 0.01; compared with the control group and #, *p* < 0.05; ##, *p* < 0.01, versus the NAFLD group.

The effects of quinoa supplementation on body weight are shown in Figure 1A. During the 6‐week intervention phase, the weight of the NAFLD group was significantly higher than that of the control group (*p* < 0.01). Body weight in the QRT and QFT groups decreased considerably compared to that in the NAFLD group at each measurement time point (*p* < 0.01). Body fat mass and content were significantly higher in the NAFLD group than in the control group (*p* < 0.01), and the body fat mass and content in the QRT and QFT groups were lower than those in the NAFLD group (*p* < 0.01). Further, the body lean mass in the NAFLD group was slightly elevated compared to the other three groups, but the body lean content was markedly reduced (*p* < 0.01) (Figure 1B,C). The QRT and QFT groups exhibited nearly identical results. In addition to weight and body fat, we measured blood glucose and insulin levels and performed HOMA‐IR (Figure 1D–F). Throughout the intervention, blood glucose levels were higher in the NAFLD group than in the control group (*p* < 0.01), whereas blood glucose levels in the QRT and QFT groups tended to be lower than those in the NAFLD group (*p* < 0.01). Insulin levels were slightly lower in the NAFLD group than in the control group; however, this difference was not statistically significant. Insulin levels were significantly elevated in the QRT and QFT groups compared with those in the control and NAFLD groups (*p* < 0.01); HOMA‐IR in the NAFLD group was higher than that in the control group (*p* < 0.01), and decreased to various degrees in the QRT and QFT groups compared to the NAFLD group, although no significant difference was observed. In summary, quinoa reduced body weight, body fat, and blood glucose levels in mice with NAFLD.

Serum metabolic parameters were measured after the 6‐week intervention. Compared to the control group, the NAFLD group showed significant upregulation of TC, TG, LDL‐C, ALT, AST, CREA, and BUN (*p* < 0.01), whereas HDL‐C levels decreased significantly in the NAFLD group (*p* < 0.01) (Figure 1G–N). The TC, TG, LDL‐C, ALT, AST, and CREA levels in the QRT and QFT groups were lower than those in the NAFLD group (*p* < 0.01), with no significant differences between the two groups.

### 3.2 Effects of Quinoa on the Histomorphological Changes of Various Tissues in NAFLD Mice

**FIGURE 2:**
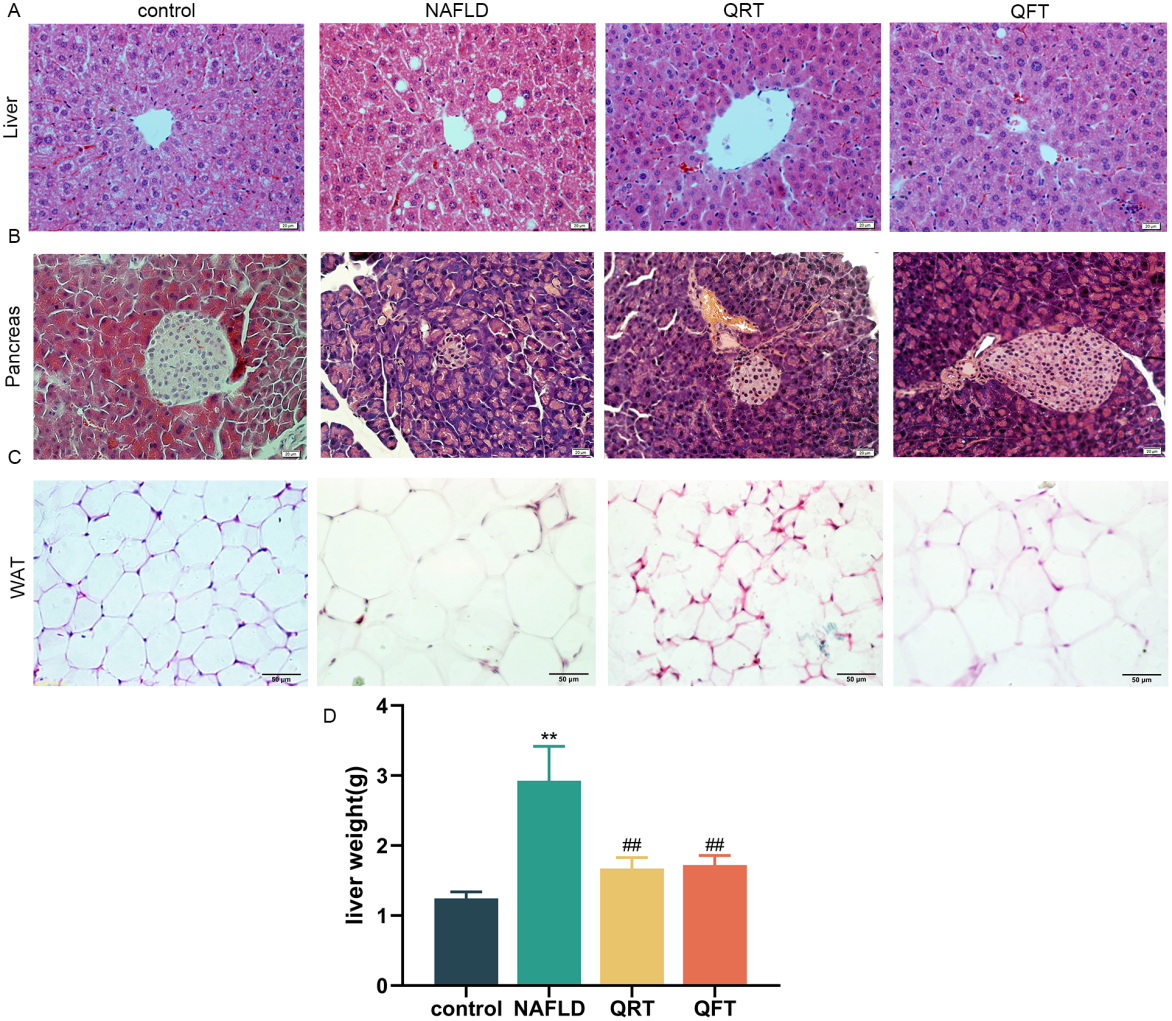
Histomorphological changes of various tissues. (A) HE staining of liver tissue sections (scale bar, 20 μm). (B) HE staining of pancreas tissue sections (scale bar, 20 μm). (C) HE staining of WAT sections (scale bar, 50 μm). (D) Changes in the liver weight. Values are expressed as the mean ± SD of five mice in each group. *, *p* < 0.05; **, *p* < 0.01; compared with the control group and #, *p* < 0.05; ##, *p* < 0.01, versus the NAFLD group, determined by ANOVA and non‐parametric test.

Histological analysis revealed excessive lipid accumulation and infiltration of inflammatory cells into the liver tissues of NAFLD mice (Figure 2A), suggesting chronic hepatocellular injury and inflammation. We observed a significant increase in the liver weight of NAFLD mice (*p* < 0.01) (Figure 2D), and hepatocyte degeneration and liver weight were significantly reduced in the QRT and QFT groups after the intervention. HE staining of the pancreatic sections (Figure 2B) showed that the islets were small, the cells in the islets were arranged irregularly, the size of the islet nuclei was irregular, and some cells showed vacuolar degeneration in the NAFLD group. The cells in the islets were arranged regularly, with clear boundaries, abundant cytoplasm, and islet nuclei of regular size and shape in the QRT and QFT groups. HE staining of white adipose tissue (WAT) (Figure 2C) showed that in the NAFLD group, the volume of adipocytes increased significantly, the size of adipocytes was uneven, the cell wall was not completely clear, and the number of intracellular lipid droplets increased. After quinoa supplementation, the WAT morphology improved to varying degrees, the cells were arranged neatly, and the cell volume decreased.

### 3.3 Quinoa May Regulate NAFLD Through the Liver via the Ras‐PLD Pathway

**FIGURE 3:**
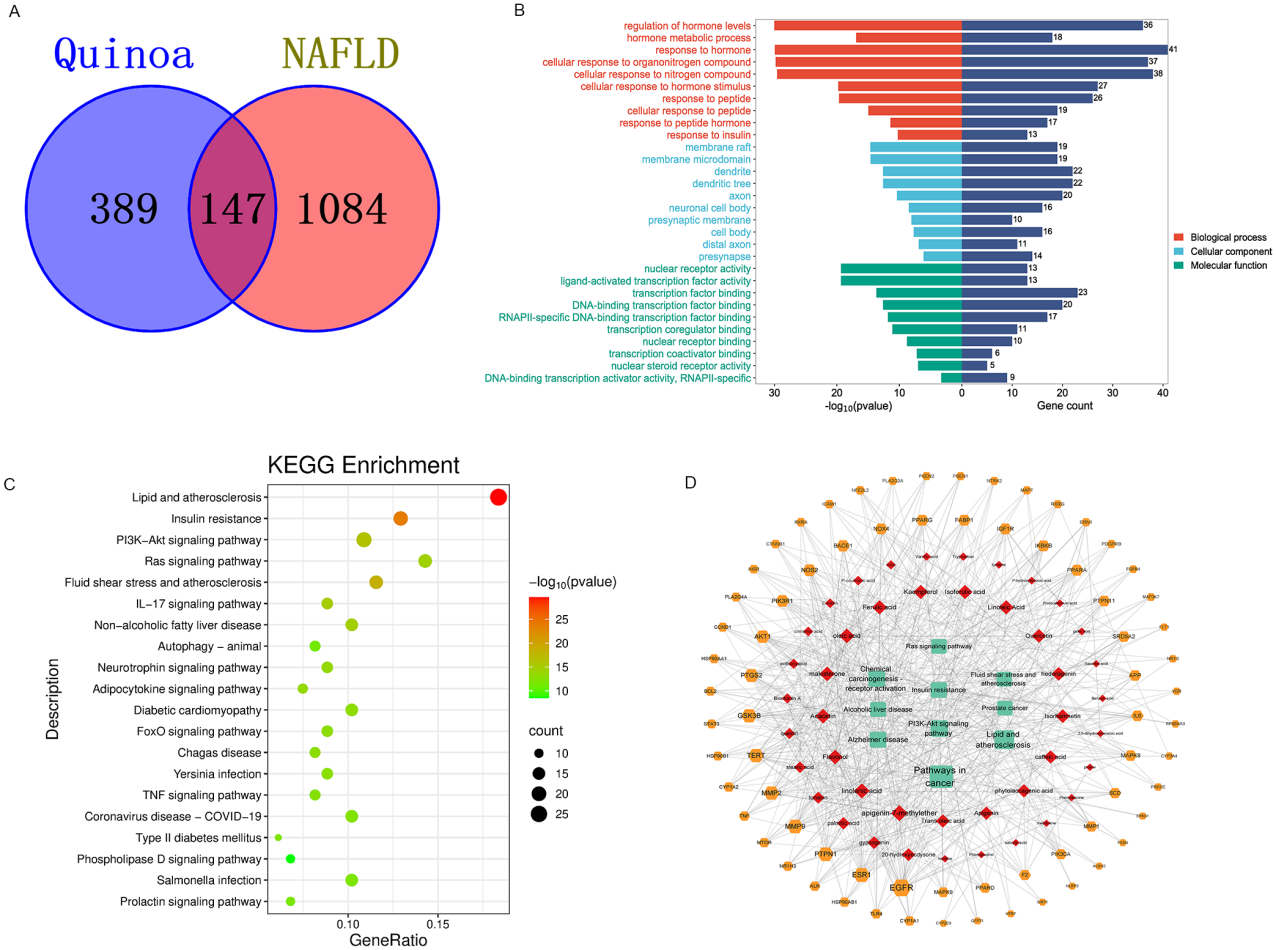
Functional characterization of quinoa against NAFLD intersecting genes. (A) Venn diagram depicting intersecting genes of quinoa and NAFLD. (B) Gene ontology analysis of intersecting genes of quinoa and NAFLD. (C) Kyoto Encyclopedia of Genes and Genomes (KEGG) pathway of intersecting genes of quinoa and NAFLD. (D) Interaction network of core components, targets and signaling pathways of quinoa against NAFLD. Red nodes represent quinoa active components, orange nodes represent potential signal pathways, green nodes represent potential targets, and wires represent interactions between the three. The larger the node area and the darker the color, the greater the impact on NAFLD.

A network pharmacology analysis was performed to elucidate the mechanism of action of quinoa in NAFLD. A total of 118 chemical components of quinoa were identified through a literature review; subsequently, 72 potentially active components were identified following intestinal absorption and drug‐like screening. A total of 536 quinoa and 1231 disease targets were identified via network pharmacological screening, and an overlap of quinoa genes with NAFLD targets revealed 147 intersecting genes between quinoa and NAFLD (Figure 3A). The 147 intersecting genes were subjected to GO enrichment analyses, which included 1570 biological processes, 97 cell compositions, and 159 molecular functions (Figure 3B). The results showed that the biological effects of quinoa in treating NAFLD mainly included the regulation of hormone levels, hormone metabolic processes, and responses to hormones. The cellular composition mainly included membrane rafts, membrane microdomains, and dendrites. The molecular functions included binding to nuclear receptors, ligand‐activated transcription factors, and transcription factors. KEGG signaling pathway enrichment resulted in 180 signaling pathways (Figure 3C). The more abundant targets are the Ras signaling pathway, lipid and atherosclerosis, PI3K‐Akt signaling pathway, autophagy‐animal, and phospholipase D signaling pathway. We used Cytoscape 3.9.1 to build a quinoa component‐target‐pathway network diagram, which comprised 122 nodes and 576 edges (Figure 3D). The degree, betweenness, and closeness centralities of apigenin‐7‐methylether were 18, 0.029, and 0.426, respectively, suggesting that apigenin‐7‐methylether is the main compound affecting the mechanism of action of quinoa in NAFLD, followed by acacetin (16, 0.021, and 0.42) and linolenic acid (16, 0.016, and 0.403). This indicates that the mechanism of action of quinoa in treating NAFLD is based on the synergistic effects of multiple compounds, genes, and targets. Apigenin‐7‐methylether, acacetin, linolenic acid rank high in degree values. This indicates that the mechanism of action of quinoa in treating NAFLD is based on the synergistic effects of multiple compounds, genes, and targets.

**FIGURE 4:**
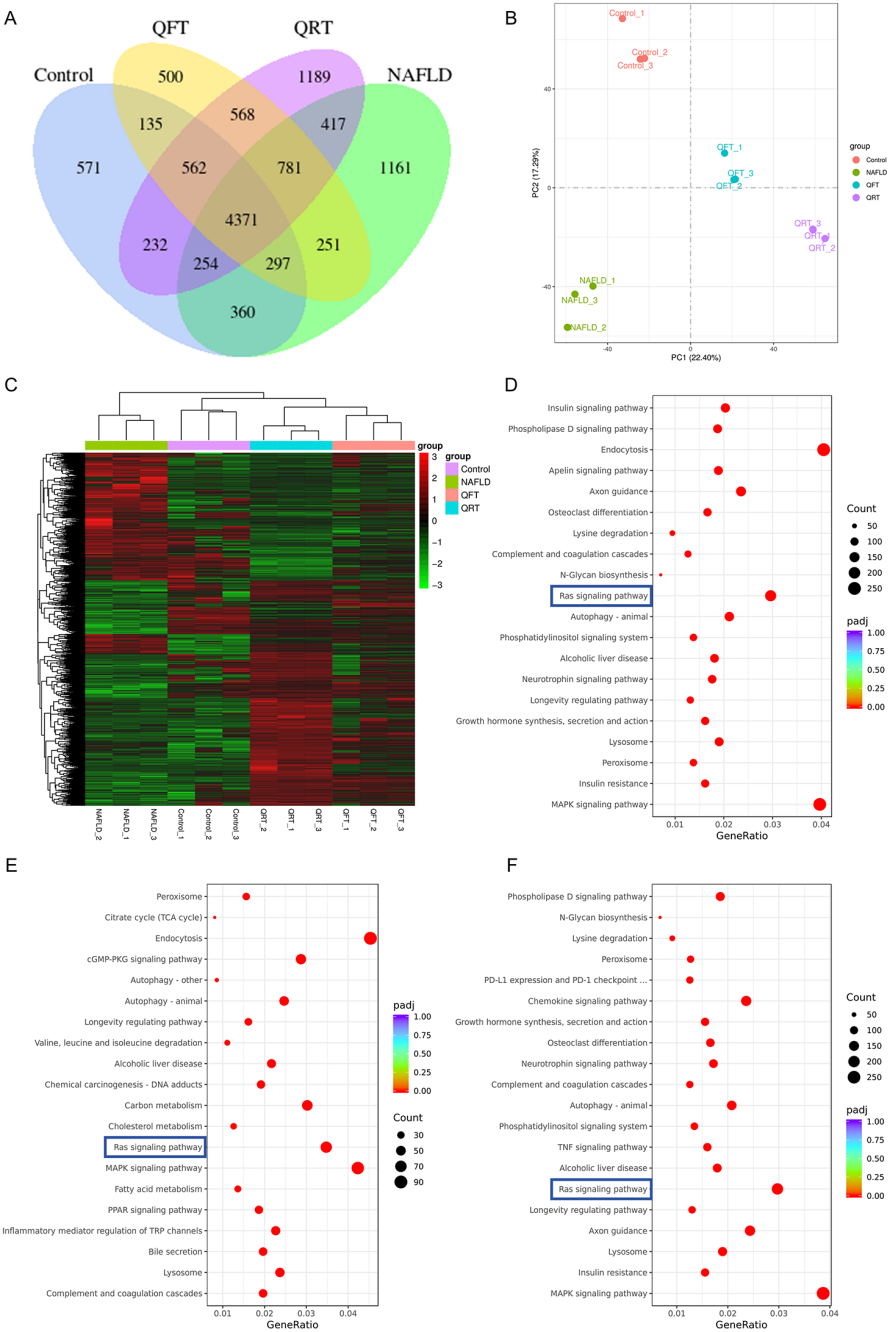
Quinoa's effect on the transcriptome changes of NAFLD mice. (A) Venn diagram depicting intersecting genes in each group. (B) PCA plot of transcriptomes of all groups. (C) Heat map of cluster analysis of differentially expressed genes in each group. (D) KEGG pathway enrichment analysis of differentially expressed genes between Control and NAFLD groups. (E) KEGG pathway enrichment analysis of differentially expressed genes between NAFLD and QRT groups. (F) KEGG pathway enrichment analysis of differentially expressed genes between NAFLD and QFT groups.

The results of transcriptome analysis indicated a considerable difference between each group (Figure 4A). PCA (principal component analysis) showed that the four groups had distinct gene expression profiles (Figure 4B). The clustering heat map showed different clustering patterns between the Control group and the NAFLD group, while clustering patterns were similar between the Control group and the QFT group (Figure 4C). The KEGG pathway analysis results revealed that NAFLD was highly associated with the RAS signaling pathway, autophagy‐ animal, and phospholipase D signaling pathway (Figure 4D). Notably, differential genes between the NAFLD group and two treatment groups of quinoa were also enriched in the RAS signaling pathway and autophagy‐ animal (Figure 4E,F).

**FIGURE 5:**
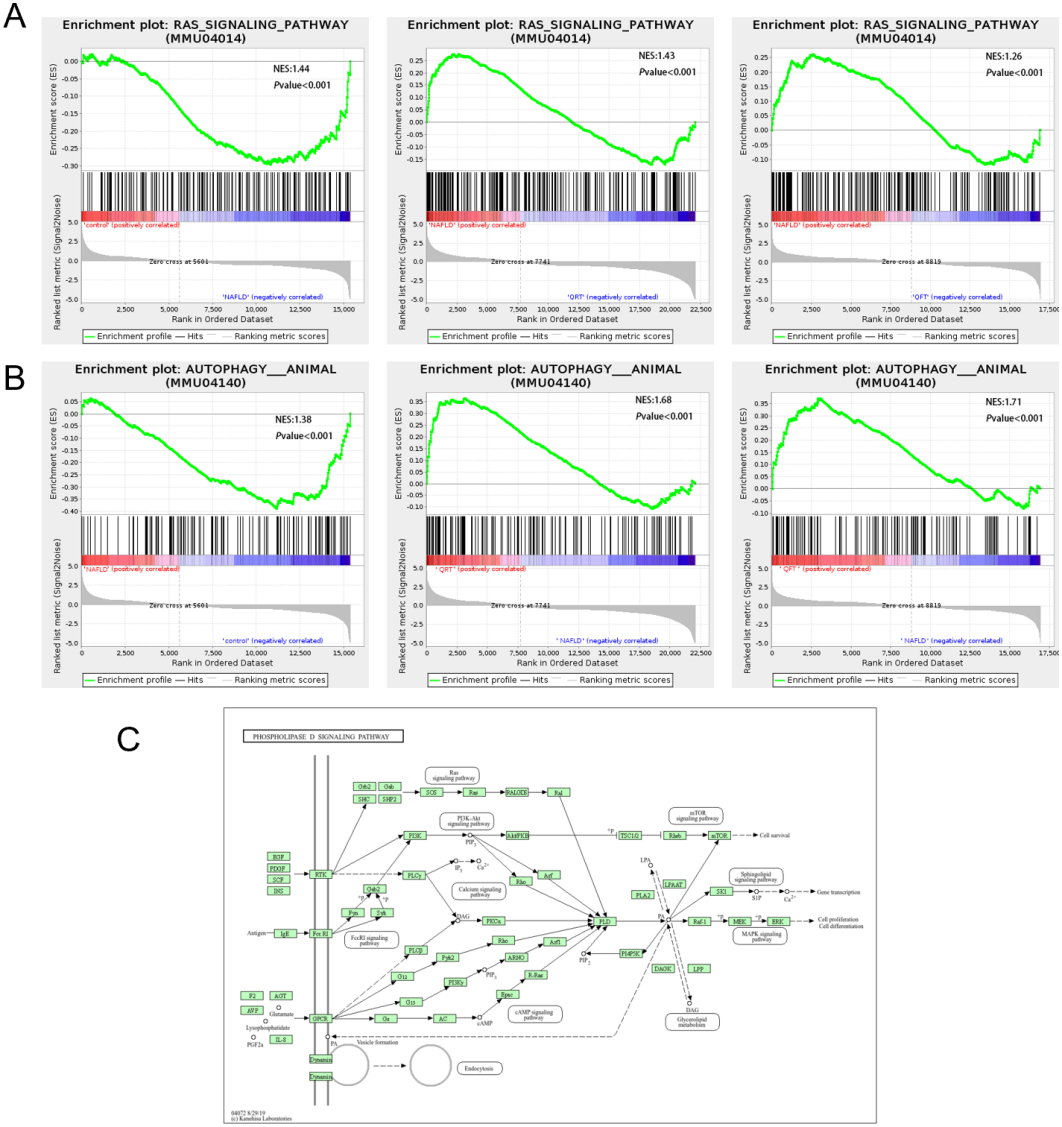
Quinoa regulates the Ras‐PLD signaling pathway and autophagy in NAFLD mice. (A) Analysis of GSEA results of Ras signaling pathway among groups. (B) Analysis of GSEA results of autophagy‐animals among groups. (C) Schematic diagram of phospholipase D signaling pathway.

To determine whether the significantly enriched signaling pathways in NAFLD were activated or inhibited by quinoa intervention, GSEA analysis was conducted. The results showed that after quinoa intervention, the RAS signaling pathway was inhibited in NAFLD mice (Figure 5A), and the autophagy‐animal pathway was activated (Figure 5B). Interestingly, the phospholipase D signaling pathway is also a key node in the RAS pathway (Figure 5C).

### 3.4 Quinoa May Regulate the Ras‐PLD Signaling Pathway and Autophagy Related Protein Expression in the Liver of NAFLD Mice

**FIGURE 6:**
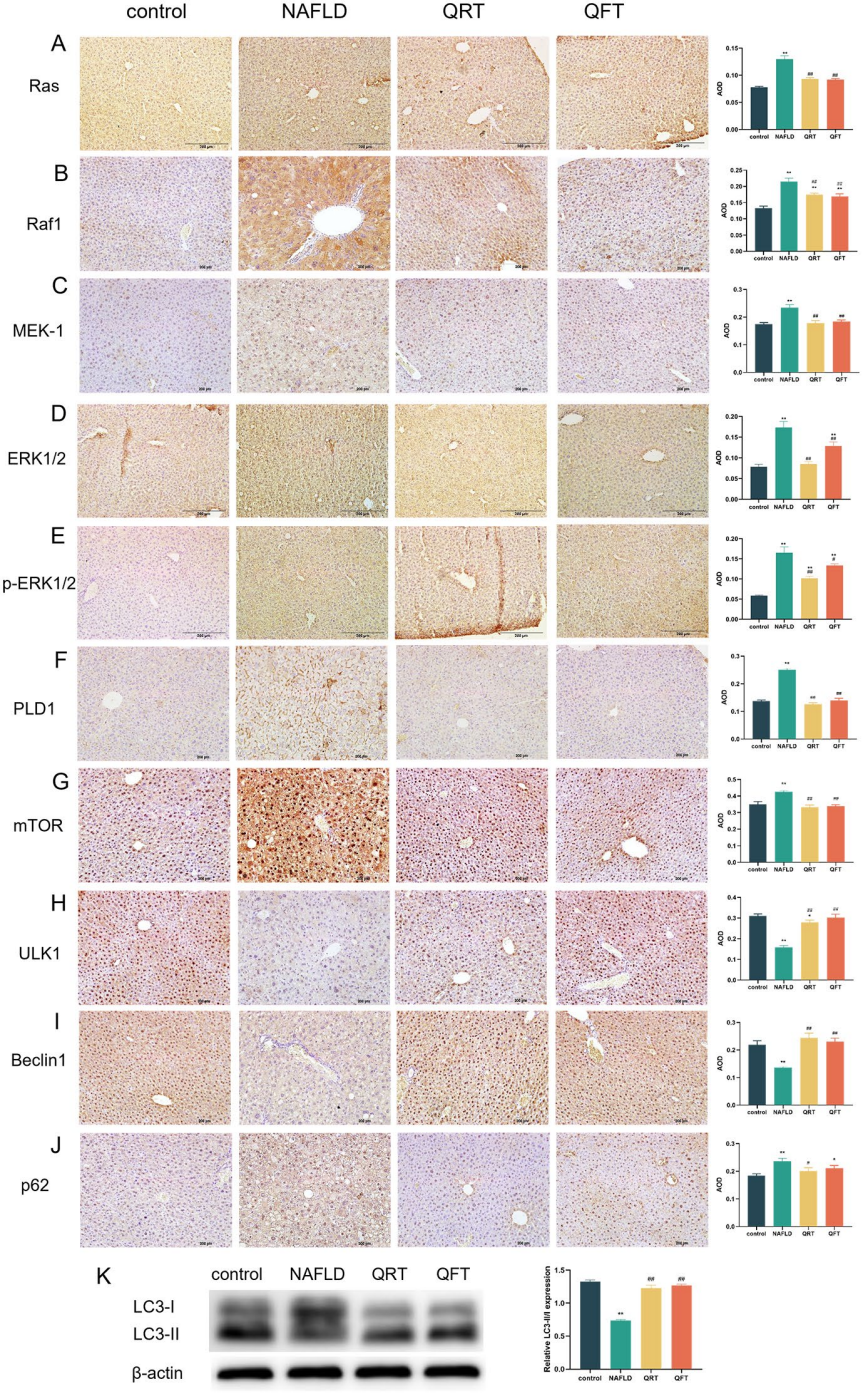
Quinoa may regulate the Ras‐PLD signaling pathway and autophagy‐related protein expression in the liver. (A–J) IHC of Ras, Raf1, MEK‐1, ERK1/2, p‐ERK1/2, PLD1, mTOR, ULK1, Beclin1, p62, and quantification of mean optical density (AOD) values in the liver using Image‐J. (K) Representative western blot of LC3B in the liver and results of western blot analysis quantitatively evaluated by Image J. *, *p* < 0.05; **, *p* < 0.01; compared with the control group and #, *p* < 0.05; ##, *p* < 0.01, versus the NAFLD group.

The above results indicate that the Ras‐PLD signaling pathway and autophagy play important roles in NAFLD. Therefore, we investigated the role of quinoa in this pathway. Compared with the control group, the expression levels of Ras, Raf1, MEK‐1, ERK1/2, p‐ERK1/2, PLD1, mTOR, and p62 in the liver tissue of NAFLD group mice were significantly increased (*p* < 0.01), while the expression levels were significantly decreased after quinoa intervention. The expression of ULK1 and Beclin1 in the liver tissue of NAFLD group mice was significantly reduced (*p* < 0.01), while the expression level was significantly increased after quinoa intervention. Meanwhile, the LC3‐II/I ratio in the NAFLD group was markedly decreased compared to the control group, while the QRT and QFT groups showed a significant upregulation trend compared to the NAFLD group (Figure 6). The results indicate that quinoa has an impact on NAFLD, which may be related to the Ras‐PLD signaling pathway and autophagy.

### 3.5 Quinoa May Regulate the Expression of Ras‐PLD Signaling Pathway Related Genes in the Liver of NAFLD Mice

**FIGURE 7:**
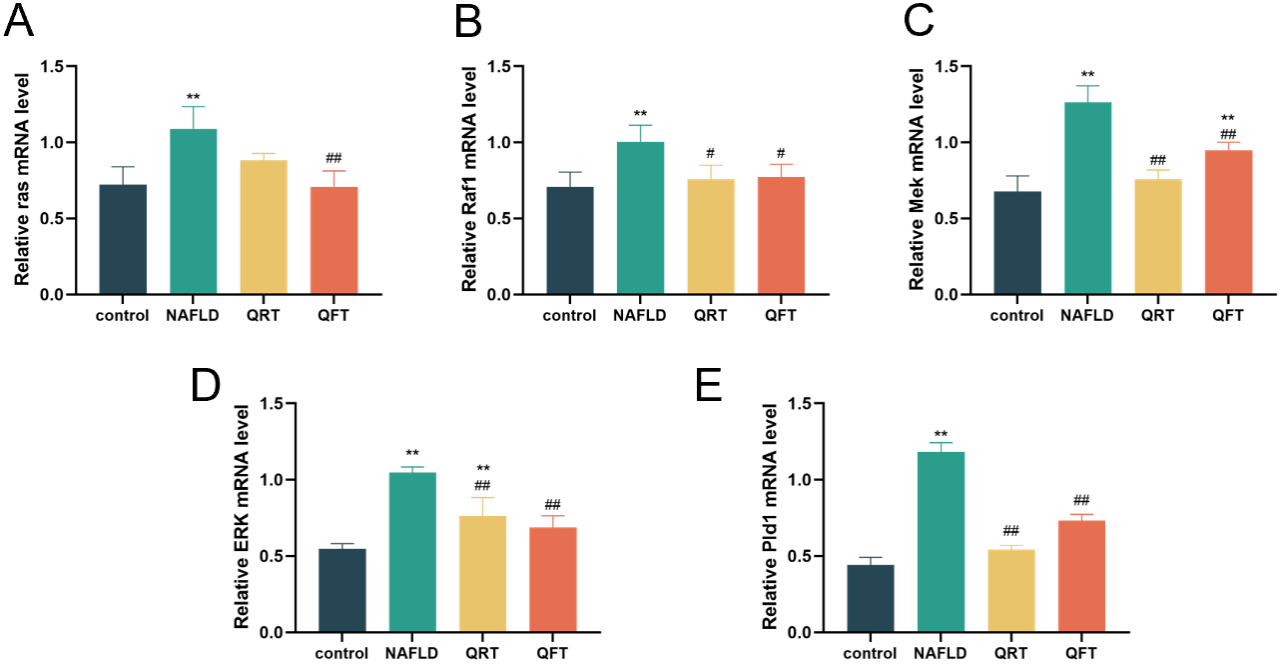
Quinoa may regulate the expression of Ras‐PLD signaling pathway related genes in the liver of NAFLD mice. (A) The mRNA level of ras in the liver. (B) The mRNA level of Raf1 in the liver. (C) The mRNA level of Mek in the liver. (D) The mRNA level of ERK in the liver. (E) The mRNA level of Pld1 in the liver. *, *p* < 0.05; **, *p* < 0.01; compared with the control group and #, *p* < 0.05.

To further elucidate the Ras‐PLD signaling pathway and autophagy mechanism in the treatment of NAFLD with quinoa, the mRNA levels of ras, Raf1, Mek, ERK, and Pld1 in liver tissue were evaluated by RT‐qPCR (Figure 7). The results showed that the mRNA levels of ras, Raf1, Mek, ERK, and Pld1 in the liver tissue of NAFLD group mice were significantly higher than those in the control group (*p* < 0.01), while the mRNA levels significantly decreased after quinoa intervention (*p* < 0.05), which were consistent with the results of IHC experiments.

### 3.6 Quinoa Affected the Expression of HNF4A, ACOX2, and Glucagon in NAFLD Mice

**FIGURE 8:**
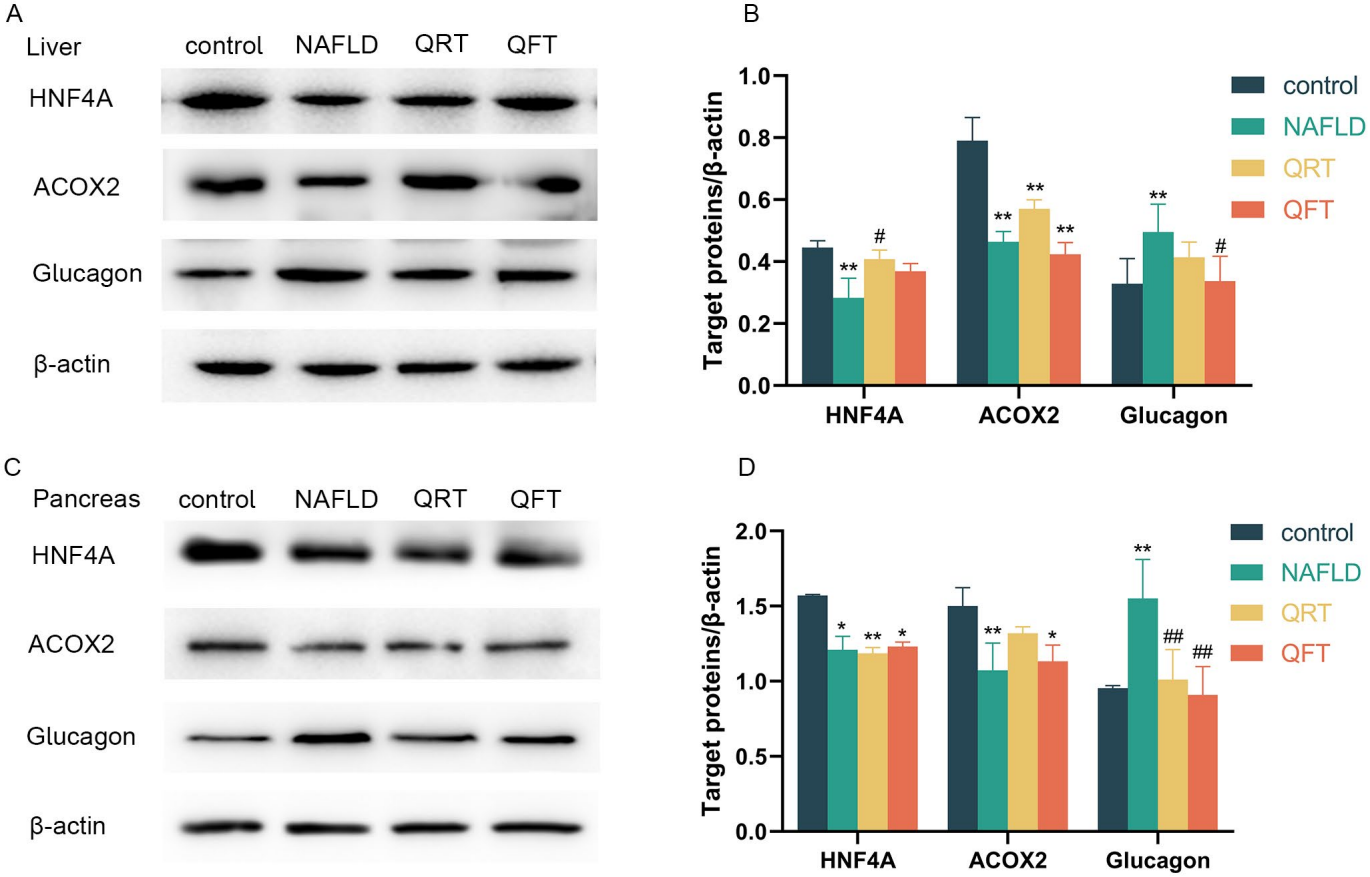
Quinoa may regulate HNF4A, ACOX2 and glucagon in the liver and pancreas tissues of NAFLD mice. (A) Representative western blot of HNF4A, ACOX2 and glucagon in the liver. (B) Quantitative assessment of the western blot analysis results of HNF4A, ACOX2 and glucagon in the liver by Image J. (C) Representative western blot of HNF4A, ACOX2 and glucagon in the pancreas. (D) Quantitative assessment of the western blot analysis results of HNF4A, ACOX2 and glucagon in the pancreas by Image J. *, *p* < 0.05; **, *p* < 0.01; compared with the control group and #, *p* < 0.05; ##, *p* < 0.01, versus the NAFLD group, determined by ANOVA and non‐parametric test.

We evaluated the expression of HNF4A in liver and pancreatic tissues, and the results showed that compared with the control group, the expression of HNF4A in the NAFLD group showed a significant downward trend (Figure 8). In the QRT and QFT groups, the expression of HNF4A increased in the liver of mice, and slightly increased in the pancreas of mice in the QFT group. The expression of ACOX2 in the liver and pancreas of NAFLD group mice was lower than that in the control group, and slightly increased after quinoa intervention. The expression of glucagon in the liver and pancreatic tissues of NAFLD group mice was significantly higher than that of the control group (*p* < 0.01), while the expression of glucagon in QRT and QFT group mice was lower than that of the NAFLD group.

## 4 Discussion

Quinoa can prevent several lifestyle‐related diseases such as obesity, diabetes, and NAFLD. Quinoa and its main components have been confirmed to promote healthy blood lipid levels (Mithila and Khanum 2015), and regulate glucose and lipid metabolism (Cao et al. 2020). In this study, we demonstrated the effect of a quinoa diet on NAFLD alleviation. The quinoa diet reduced body weight and body fat in NAFLD mice, with elevations typically occurring prior to the onset and initial stages of NAFLD. Development of hepatic insulin resistance may redirect glucose into lipogenic pathways and further promote NAFLD (Loomba et al. 2021); however, the quinoa diet reduced blood glucose and improved impaired glucose tolerance and insulin resistance in NAFLD mice, implying that quinoa may inhibit lipogenesis and exacerbation of NAFLD. It is interesting that the NAFLD group showed a significant increase in HOMA‐IR with hyperglycemia, but no significant increase in insulin levels. This suggests that the pathological process has progressed beyond compensatory hyperinsulinemia and progressed to pancreatic β cell dysfunction and failure. The QRT and QFT groups were able to maintain higher insulin levels and improve pancreatic islet morphology, which jointly confirmed their protective effects on beta cells and delayed the progression of functional failure from both functional and structural perspectives (Chen et al. 2021; Brereton et al. 2016). Moreover, quinoa showed major regulatory effects on biochemical indicators closely associated with glucose‐lipid metabolism and NAFLD, including TC, TG, and LDL‐C. We also found that quinoa supplementation improved the pathomorphological changes in WAT and reduced adipocyte size in NAFLD mice. Quinoa supplementation has been reported to reduce adipose tissue development in mice fed a high‐fat diet (Foucault et al. 2014); therefore, we speculated that one of the mechanisms underlying the anti‐NAFLD effects of quinoa is the regulation of lipid metabolism via multiple pathways.

Network pharmacology analysis showed that GO enrichment results indicated that the biological effects of quinoa were concentrated in hormone metabolism regulation (such as insulin, glucagon axis) and nuclear receptor binding, which was highly consistent with the pathological characteristics of hormone resistance and lipid metabolism disorders in NAFLD (Grander et al. 2023). KEGG pathway enrichment showed that the Ras signaling pathway, phospholipase D (PLD) signaling pathway, and autophagy pathway were key hubs. Acacetin may regulate lipid metabolism through the AMPK pathway, which is consistent with the results of this study that quinoa reduced TC and TG (Liou et al. 2022).

Transcriptome PCA and cluster analysis showed that the gene expression profile of the quinoa treatment group (QFT) tended to approach that of the control group, indicating that it could reverse NAFLD‐related gene abnormalities. KEGG pathway analysis confirmed that the Ras signaling pathway was significantly activated in the NAFLD group, while it was significantly inhibited after quinoa intervention. Overactivation of the Ras‐PLD pathway can induce hepatic adipogenesis and inhibit autophagy by promoting phosphorylation of extracellular signal‐regulated kinase 1/2 (ERK1/2) (He et al. 2016). In this study, the protein expression of Ras, Raf1, MEK, ERK p‐ERK1/2, and PLD1 significantly increased in the liver tissue of NAFLD mice, while quinoa intervention showed a decreasing trend. The changes in mRNA levels were consistent with protein expression, indicating that quinoa may activate autophagy by inhibiting the core molecules of the Ras‐PLD pathway, thereby alleviating liver steatosis.

Dysfunction of autophagy is a key factor in the progression of NAFLD (Qian et al. 2021; Ren et al. 2024), and the decreased expression of ULK1 and Beclin1, as core proteins in the initiation stage of autophagy, can lead to lipid droplet clearance disorders (Martinez‐Lopez and Singh 2015). This study found that ULK1, Beclin1 and LC3‐II/I ratio expression were significantly downregulated in the liver tissue of NAFLD mice, while p62 (an autophagy degradation marker) was significantly accumulated (Liu et al. 2016), indicating that the autophagy pathway was inhibited. After intervention with quinoa, the expression of ULK1, Beclin1 and LC3‐II/I ratio were upregulated and p62 was reduced, indicating the restoration of autophagic activity (Yang et al. 2024). It is worth noting that the PLD signaling pathway is a downstream node of the Ras pathway, and its product phosphatidic acid (PA) can inhibit autophagy by activating mammalian rapamycin target protein (mTOR) (Jang et al. 2014). The inhibition of PLD1 by quinoa may be relieved by blocking the mTOR/p62 pathway, which is consistent with the activation of the autophagy pathway shown by GSEA.

This study found that the expression of HNF4A in the liver and pancreas tissues of NAFLD mice was significantly downregulated, while quinoa intervention partially restored its levels, which may be related to abnormal activation of the Ras pathway. HNF4A is an orphan transcription factor member of the nuclear receptor superfamily, expressed in the liver and pancreas (Ihara et al. 2005). HNF4A not only regulates lipid transporters and lipoproteins, but also controls insulin resistance induced pancreatic cell mass expansion and regulates glucocorticoid activity in the liver (Barth et al. 2022; Hunter et al. 2022). HNF4A, as a transcription factor, can inhibit Ras‐ERK1/2 signaling to maintain lipid homeostasis. Its absence may lead to overactivation of the Ras pathway and promote liver fat production (Delire and Stärkel 2015). ACOX2 is an enzyme involved in peroxisome bile acid synthesis and branched chain fatty acid degradation, primarily expressed in the liver and playing a critical role in liver metabolic homeostasis (Zhang et al. 2022). ACOX2 dysfunction may lead to the accumulation of C27 bile acids, while idiopathic bile acid intermediates may cause oxidative and endoplasmic reticulum stress during liver cell hypertransaminase syndrome (Winther‐Sørensen et al. 2020). Our results are consistent with this mechanism, as the expression of ACOX2 in the liver of NAFLD mice is reduced, while the levels of ALT and AST are significantly increased. Quinoa upregulates ACOX2 in the liver and reduces ALT and AST levels, indicating that ACOX2 may be another potential target for treating NAFLD liver dysfunction. At the same time, the decreased expression of ACOX2 in the NAFLD group suggests impaired function of peroxisomes, which are involved in fatty acid β‐oxidation. The decreased activity of this enzyme may inhibit autophagy initiation protein ULK1/Baclin1 by accumulating oxidative stress products, while phospholipase D (PLD)‐mediated generation of phosphatidic acid (PA) can further inhibit autophagy by activating mTOR (Shen et al. 2024). Glucagon is a hyperglycemic substance in the pancreas that increases liver glucose production and regulates amino acid metabolism in the liver (Perry et al. 2020). Glucagon stimulates hepatic gluconeogenesis by increasing hepatic triglyceride lipase activity and hepatic lipolysis. According to reports, fasting glucagon and amino acid levels are elevated in NAFLD patients, which may be due to amino acid clearance disorders and impaired liver function of glucagon hormones (Winther‐Sørensen et al. 2020; Pedersen et al. 2020). In our experiment, the expression of glucagon increased in the NAFLD group, which may be due to high fat degeneration. It is worth noting that the levels of glucagon in the liver and pancreas were significantly downregulated in the QRT and QFT groups. Therefore, we speculate that quinoa affects glucose and lipid metabolism through glucagon regulation. In summary, quinoa exerts improvement effects through multiple pathways and targets.

We have systematically suggested that quinoa may improve NAFLD by regulating the Ras/PLD signaling pathway and autophagy process through the integration of network pharmacology, transcriptomics, and in vivo experiments. However, this study still has some limitations. Although this multi‐omics and multi‐level association analysis provides strong clues, ultimately establishing a direct causal relationship still relies on rescue experiments. Therefore, our next work will focus on clearly verifying the core roles of Ras/PLD signaling and autophagy in this process through in vitro and in vivo rescue experiments. This not only makes up for the shortcomings of this study, but also greatly deepens our understanding of the intervention effects of quinoa.

## 5 Conclusion

Our experimental results showed that quinoa could be beneficial in NAFLD treatment. The results in the QRT group were slightly better than those in the QFT group, but the differences between them were not statistically significant, which may be due to this difference in dosage. In the present study, we confirmed that quinoa can improve glucose and lipid metabolism abnormalities and insulin resistance by regulating the Ras‐PLD signaling pathway and autophagy, thereby alleviating NAFLD.

## Author Contributions

Y.Z.: conceptualization, methodology, formal analysis, writing – original draft, visualization. Z.L.: project, writing – review and editing. J.L.: conceptualization, formal analysis, supervision. X.Z.: data curation. R.L.: visualization. X.L.: formal analysis, writing – review and editing. G.J.: supervision, project, administration, writing – review and editing. All authors read and approved the final manuscript.

## Funding

This work was supported by the Incubation Project for Major Scientific Research Programs of Xinjiang Medical University (XYD2024ZX06), the horizontal projects of Xinjiang Tianrun Dairy Co. Ltd. (BUCM‐2024‐JS‐FW‐068) and Zhongfu Boai Health Management (Beijing) Co. Ltd. (21800717172024).

## Ethics Statement

The study protocol was approved by the Animal Care and Management Committee of the Beijing University of Chinese Medicine (ethical batch number: BUCM‐2019041501‐200J). All manipulations were at the request of the guidelines of the Animal Care Committee.

## Conflicts of Interest

The authors declare no conflicts of interest.

## Data Availability

The data that support the findings of this study are available from the corresponding author upon reasonable request. Raw sequence data of microbiota and transcriptome that support the findings in our study have been deposited into NCBI's Sequence Read Archive under accession number PRJNA1103418 and PRJNA1103733.
